# Admission glucose as a prognostic marker for all-cause mortality and cardiovascular disease

**DOI:** 10.1186/s12933-022-01699-y

**Published:** 2022-11-26

**Authors:** Catarina Djupsjö, Jeanette Kuhl, Tomas Andersson, Magnus Lundbäck, Martin J. Holzmann, Thomas Nyström

**Affiliations:** 1 Department of Medicine, Karolinska Institutet, Stockholm, Sweden; 2 Heart and Vascular Theme, Karolinska University Hospital, Stockholm, Sweden; 3 Division of Medicine, Danderyd University Hospital, Stockholm, Sweden; 4 Institute of Environmental Medicine, Karolinska Institutet, Stockholm, Sweden; 5 Center for Occupational and Environmental Medicine, Stockholm County Council, Stockholm, Sweden; 6 Department of Clinical Sciences, Division of Cardiovascular Medicine, Danderyd University Hospital, Karolinska Institutet, Stockholm, Sweden; 7 Functional Area of Emergency Medicine, Karolinska University Hospital, Stockholm, Sweden; 8 Department of Clinical Science and Research, Karolinska Institutet, Stockholm, Sweden; 9 Division of Internal Medicine at Södersjukhuset, Stockholm, Sweden

**Keywords:** Random plasma glucose, Metabolic status, Cardiovascular disease, Mortality, Emergency department

## Abstract

**Background:**

Diabetes and prediabetes are known risk factors for cardiovascular disease and associated with increased mortality risk. Whether patients with a random elevated blood glucose level but no history of diabetes are at a higher mortality and cardiovascular risk is not entirely known.

**Methods:**

A retrospective cohort study where patients (18–80 years) with no history of diabetes between 2006 and 2016 attending the emergency department (ED) in Sweden were included. Based on the first (index) blood glucose level patients were categorized into four groups: hypoglycemia (< 3.9 mmol/L), normal glucose tolerance (NGT) (3.9–7.8 mmol/L), dysglycemia (7.8–11.1 mmol/L), and hyperglycemia (> 11.1 mmol/L). Data was collected from four nationwide registers (National Patient Register, National Cause of Death Register, Prescribed Drug Register and Statistics Sweden). Cox regression was used to calculate adjusted hazard ratios (HR) with 95% confidence intervals (CI) for all-cause mortality and cardiovascular outcomes using NGT as reference.

**Results:**

618,694 patients were included during a mean follow-up time of 3.9 years. According to the index blood glucose level: 1871 (0.3%) had hypoglycemia, 525,636 (85%) had NGT, 77,442 (13%) had dysglycemia, and 13,745 (2%) patients had hyperglycemia, respectively. During follow-up 44,532 (7.2%) deaths occurred. After multiple adjustments, mortality risk was highest in patients with hypoglycemia HR 2.58 (2.26–2.96) followed by patients with hyperglycemia HR 1.69 (1.63–1.76) and dysglycemia HR 1.16 (1.13–1.19). Risk for cardiovascular events: i.e., myocardial infarction, stroke and heart failure, were highest among patients with hyperglycemia HR 2.28 (2.13–2.44), HR 1.62 (1.51–1.74) and HR 1.60 (1.46–1.75), respectively.

**Conclusion:**

Patients with disturbed blood glucose level at ED admission have a higher mortality risk than patients with NGT. Patients with hyperglycemia have almost a two folded increased long-term mortality risk and more than a doubled risk for cardiovascular events compared to patients with NGT.

**Supplementary Information:**

The online version contains supplementary material available at 10.1186/s12933-022-01699-y.

## Background

Patients attending the emergency department (ED) may have elevated blood glucose, without having diabetes, which is suggested to be due to stress [1] and explained by different physiological mechanisms compared to prediabetes and type 2 diabetes [2]. Studies have shown that an elevated admission blood glucose level, in correlation with a specific condition, such as: myocardial infarction, stroke, heart failure or pneumonia is associated with a higher in-hospital mortality, increased length of hospital stay, and a higher rate of in-hospital complications [3–12]. Long-term mortality risk and risk of cardiovascular events and its association to a random blood glucose level at ED admission has previous been studied in different health interview surveys [13–15]. It was recently shown that in an unselected small cohort of patients admitted to an acute general medical ward, patients with one elevated blood glucose, no matter the cause of admission, had a higher mortality after one year, but not after two years [16].

Studies demonstrate that cardiovascular complications from diabetes can appear at the same time as diabetes is diagnosed, as previously undiagnosed diabetes and impaired glucose tolerance are common in patients with acute myocardial infarction [17]. Approximately two-thirds of patients admitted for acute myocardial infarction have unknown disturbances of glucose metabolism detected by either glycated hemoglobin A1c (HbA1c) at admission, oral glucose tolerance test (OGTT), or a fasting blood glucose (FPG) concentration [18]; with similar results after 3 months of follow-up [19]. The same picture was found in elderly patients with stroke, whereas HbA1c and OGTT revealed that almost two-thirds of the patients had unknown disturbances of glucose metabolism at the time of their stroke, although at three months follow-up the disturbances of glucose levels were less noticeable [20].

The association between elevated random blood glucose levels at hospital admission and short-term outcome is well studied [7, 12]. Whether a simple random blood glucose level also can predict outcomes such as death and cardiovascular complications in the long run is less studied in a European population [14, 15]. The aim of the present study is to investigate the association of a random blood glucose level at ED admission and mortality risk and cardiovascular events, i.e., myocardial infarction, stroke and heart failure, in patients with no history of diabetes.

## Methods

### Study design

This is a retrospective cohort study. Study reporting followed the STROBE guidelines for observational studies using routinely collected data [21]. The study complied with the Declaration of Helsinki and was approved by the regional research ethics committee in Stockholm, Sweden (2018/1089-31, 2019–02339 and 2020–05925).

### Study population

From 2006 to 2016, all patients 18–80 years of age, attending the ED in four hospitals in Stockholm, Sweden (i.e., Karolinska University Hospital Huddinge, Karolinska University Hospital Solna, Danderyd University Hospital and Södersjukhuset) and three hospitals in Gothenburg, Sweden (i.e., Sahlgrenska Hospital, Östra Hospital and Mölndal Hospital), were included.

Only the first visit to the ED during the study period was accepted as an inclusion to the study. History of diabetes were determined by controlling which patients had collected antidiabetic treatment from the pharmacy according to the prescribed drug register (PDR) before admission [22]. Furthermore, patients with known diabetes (ICD 10 codes: E10-E14) from the national patient register (NPR) were also excluded.

Patient data was collected by individual-level data-linking using the unique personal identity number assigned to all persons living in Sweden [23, 24]. Baseline characteristics, hospital stay and medical data were collected from the national patient register (NPR) [25]. In collecting baseline characteristics, both primary and secondary diagnoses were accepted as medical history to ensure that the patients' medical backgrounds were fully identified. The index diagnosis, i.e., the reason why the patients were attending the ED in the first place was not included in the medical background. Information regarding mortality was collected from the national cause of death register [26] and cardiovascular mortality was defined as the primary diagnosis from the same register. For the definition of myocardial infarction, stroke and heart failure, ICD diagnosis codes from the NPR were used (Additional file 1: Table S1).

Information regarding revascularization (i.e., percutaneous coronary intervention [PCI]) and coronary artery bypass grafting [CABG]) was collected from NPR (Additional file 1: Table S1). Information about medication/drugs was collected from PDR [27], and all drugs collected 365 days before the visit to the ED were accepted. Information regarding socioeconomic status and education were collected from statistics Sweden (Statistiska Centralbyrån) [28].

### Exposure

Depending on the result from the random blood glucose level at admission to the ED, patients were divided into four glucose tolerance groups according to the American Diabetes Association 2021 [29].

- Hypoglycemia—Random plasma glucose value at < 3.9 mmol/L.
- Normal glucose tolerance (NGT)—Random plasma glucose level at ≥ 3.9 to < 7.8 mmol/L.
- Dysglycemia—Random plasma glucose level ≥ 7.8 to < 11.1 mmol/L.
- Hyperglycemia—Random plasma glucose value ≥ 11.1 mmol/L.

All hospitals used plasma glucose as the measurement method except for the hospitals in Gothenburg where venous blood glucose was measured instead. The formula “plasma glucose = venous blood glucose x 1.11” was used to equalize all the measurements in the current study [30].

### Outcomes

The primary outcome was all-cause mortality. Secondary outcomes were cardiovascular events, i.e., cardiovascular mortality, myocardial infarction, stroke and hospitalization due to heart failure.

### Statistical methods

Patient characteristics were described using frequencies and percentages for categorical variables and means and standard deviations (SD) for continuous variables. For each outcome the person-time in years which was contributed by each patient, was calculated from the date of attending the ED to the date of death, myocardial infarction, stroke, heart failure or the end of the follow-up (31st December 2016). Separately for all outcomes, we calculated the crude incidence rates and 95% confidence intervals (CIs) by the following random blood glucose categories: hypoglycemia, NGT, dysglycemia and hyperglycemia. We used Cox regression to estimate the hazards ratios (HR) with NGT as the reference category. In the Cox models we used hospital as stratification variable and adjusted for visit date, age and sex. Thereafter we adjusted for all the remaining variables listed in Table 1 as covariates except for glucose. Finally, as a sensitive analysis, we also adjusted markers of acute stress, i.e., white blood cell (WBC) count. Fine and Gray method were used in Cox model when adjusting for competing risk [31]. Data management and statistical analyses were performed using SAS 9.4 for Windows (SAS Institute Inc) and R 4.1 (www.R-project.org).

**Table 1:** Baseline characteristics in 618,694 patients attending the emergency department

Variables	All	Hypoglycemia < 3.9 mmol/L	NGT 3.9–7.7 mmol/L	Dysglycemia 7.8–11.1 mmol/L	Hyperglycemia > 11.1 mmol/L
n	618,694	1871	525,636	77,442	13,745
Age, Mean (SD)	47.6 (17.9)	39.6 (16.9)	46.2 (17.7)	56.1 (16.6)	57.2 (15.8)
Men, n (%)	299,151 (48.4)	778 (41.6)	247,466 (47.1)	42,510 (54.9)	8397 (61.1)
*Comorbidities*					
Hypertension, n (%)	47,340 (7.7)	105 (5.6)	36,009 (6.9)	9514 (12.3)	1712 (12.5)
Atrial fibrillation, n (%)	17,169 (2.8)	44 (2.4)	13,458 (2.6)	3127 (4.0)	540 (3.9)
Coronary heart disease, n (%)	23,272 (3.8)	52 (2.8)	17,925 (3.4)	4471 (5.8)	824 (6.0)
Prior CABG, n (%)	3015 (0.5)	6 (0.3)	2442 (0.5)	486 (0.6)	81 (0.6)
Prior PCI, n (%)	7337 (1.2)	9 (0.5)	5882 (1.1)	1245 (1.6)	201 (1.5)
Prior stroke, n (%)	11,427 (1.8)	33 (1.8)	8828 (1.7)	2170 (2.8)	396 (2.9)
Peripheral arterial disease, n (%)	1152 (0.2)	8 (0.4)	832 (0.2)	245 (0.3)	67 (0.5)
COPD, n (%)	8485 (1.4)	40 (2.1)	6378 (1.2)	1678 (2.2)	389 (2.8)
CKD, n (%)	2657 (0.4)	9 (0.5)	2060 (0.4)	487 (0.6)	101 (0.7)
^a^History of malignancy, n (%)	18,473 (3.0)	36 (1.9)	13,925 (2.6)	3813 (4.9)	699 (5.1)
*Laboratory values*					
eGFR, Mean (SD)	96.4 (21.9)	100.7 (28.5)	97.7 (21.4)	87.4 (22.5)	85.5 (26.1)
> 60 ml/min, n (%)	483,942 (94.4)	1 495 (91.5)	425 360 (95.3)	49 045 (88.9)	8 042 (82.8)
30–60 ml/min, n (%)	24,636 (4.8)	72 (4.4)	17 976 (4.0)	5 194 (9.4)	1 394 (14.3)
15–30 ml/min, n (%)	2778 (0.5)	43 (2.6)	1 912 (0.4)	624 (1.1)	199 (2.0)
< 15 ml/min, n (%)	1556 (0.3)	23 (1.4)	1 148 (0.3)	303 (0.5)	82 (0.8)
Random glucose, Mean (SD)	6.5 (2.6)	3.4 (0.5)	6.0 (0.8)	8.8 (0.8)	17.1 (10.7)
*Medication at index visit*					
Statin therapy, n (%)	46,885 (7.6)	65 (3.5)	35,598 (6.8)	9609 (12.4)	1613 (11.7)
Aspirin, n (%)	40,293 (6.5)	73 (3.9)	30,670 (5.8)	8150 (10.5)	1400 (10.2)
P2Y12inhibitors, n (%)	3439 (0.6)	11 (0.6)	2655 (0.5)	671 (0.9)	102 (0.7)
Betablockers, n (%)	64,023 (10.3)	132 (7.1)	48,563 (9.2)	12,916 (16.7)	2412 (17.5)
ACE/ARB, n (%)	72,244 (11.7)	98 (5.2)	55,309 (10.5)	14,347 (18.5)	2490 (18.1)
Calcium channel-blockers, n (%)	37,065 (6.0)	60 (3.2)	27,380 (5.2)	8186 (10.6)	1439 (10.5)
OAC, n (%)	14,056 (2.3)	24 (1.3)	11,046 (2.1)	2592 (3.3)	394 (2.9)
Diuretics, n (%)	32,342 (5.2)	72 (3.8)	23,945 (4.6)	6894 (8.9)	1431 (10.4)
*Socioeconomics*					
Married, n (%)	249,471 (40.3)	500 (26.7)	206,987 (39.4)	36,032 (46.5)	5952 (43.3)
Not married, n (%)	346,973 (56.1)	1333 (71.2)	301,880 (57.4)	36,788 (47.5)	6972 (50.7)
Widowed, n (%)	22,250 (3.6)	38 (2.0)	16,769 (3.2)	4622 (6.0)	821 (6.0)
*Education*					
Primary school, n (%)	131,308 (21.2)	495 (26.5)	109,027 (20.7)	1,991 (23.2)	3795 (27.6)
Collage, n (%)	254,441 (41.1)	734 (39.2)	216,009 (41.1)	31,968 (41.3)	5730 (41.7)

ACE, angiotensin converting enzyme inhibitors; ARB, angiotensin receptor blockers; CABG, coronary artery bypass grafting, COPD, chronic obstructive pulmonary disease; CKD, chronic kidney disease; eGFR, estimated glomerular filtration rate; OAC, oral anticoagulants; PCI, percutaneous coronary intervention SD, Standard deviation; n, number of patients

^a^Any history of malignancy within 2 years prior to index-date

## Results

### Baseline characteristics

Baseline characteristics are shown in Table 1. 618,694 patients with a mean age of 47.6 (17.9) years were included, of whom 299,159 (48.4%) were men. According to the categorization after the index blood glucose level: 1871 (0.3%) had hypoglycemia, 525,636 (85%) had NGT, 77,442 (13%) had dysglycemia and 13,745 (2%) had hyperglycemia, respectively (Table 1). Patients with hyperglycemia were older and more often male compared to the other groups. The prevalence of hypertension, chronic obstructive pulmonary disease, prior stroke, and peripheral arterial disease was more common in patients with hyperglycemia. Patients with dysglycemia and hyperglycemia had the highest prevalence of atrial fibrillation, coronary heart disease, prior revascularization procedure (CABG and PCI), chronic kidney disease (CDK), and history of malignancy (i.e., any history of malignancy within 2 years prior to index-date), compared to the other groups. Patients with dysglycemia were more often treated with statin therapy, aspirin, P2Y12-inhibitors, angiotensin converting enzyme inhibitors (ACEI)/angiotensin receptor blockers (ARB) and oral anticoagulants (OAC) than the other groups.

### Early (30-day) outcomes—event, event rates and risk of mortality, myocardial infarction, stroke, and heart failure due to blood glucose level categorization

During the first 30-days a total of 4780 patients died (0.8%): 80 (4.3%) patients with hypoglycemia, 2452 (0.5%) patients with NGT, 1360 (1.8%) patients with dysglycemia and 888 (6.5%) patients with hyperglycemia, respectively. Event, event rates and HRs are all shown in Table 2.

**Table 2:** Early (30-day) event, event rates and relative risks for all-cause mortality, cardiovascular mortality, myocardial infarction, stroke and heart failure in 618,694 patients attending the emergency department

Variable	Glucose group	Event	Event rate 1000 PY (CI 95%)	Age, sex, date and hospital adjusted HR (CI 95%)	Multivariable^a^ adjusted HR (CI 95%)	Multivariable^a^ + WBC adjustments HR (CI 95%)
All-cause mortality	Hypoglycemia	80	540.5 (428.6-672.7)	14.32 (11.45–17.90)	10.51 (8.40-13.16)	10.87 (8.69–13.61)
	NGT	2452	57.1 (54.8–59.4)	1	1	1
	Dysglycemia	1360	216.8 (205.4–228.6)	2.32 (2.16–2.48)	2.10 (1.96–2.25)	2.14 (1.99–2.29)
	Hyperglycemia	888	829.9 (776.2-886.3)	8.50 (7.85–9.20)	6.95 (6.41–7.53)	7.04 (6.50–7.63)
CV mortality	Hypoglycemia	8	54.1 (23.3–106.5)	7.10 (3.53–14.28)	4.85 (2.40–9.80)	5.29 (2.62–10.67)
	NGT	511	11.9 (10.9–13.0)	1	1	1
	Dysglycemia	422	67.3 (61.0–74.0)	3.24 (2.83–3.70)	3.08 (2.69–3.51)	3.13 (2.74–3.58)
	Hyperglycemia	445	415.9 (378.1–456.4)	18.91 (16.56–21.60)	15.84 (13.85–18.12)	16.43 (14.36–18.80)
Myocardial Infarction	Hypoglycemia	7	47.3 (19.0–97.5)	0.77 (0.37–1.61)	0.79 (0.38–1.66)	0.77 (0.37–1.62)
	NGT	3824	89.6 (86.8–92.5)	1	1	1
	Dysglycemia	1606	261.0 (248.4–274.1)	1.72 (1.62–1.83)	1.72 (1.62–1.83)	1.70 (1.60–1.81)
	Hyperglycemia	624	603.5 (557.1–652.7)	3.45 (3.16–3.76)	3.42 (3.14–3.73)	3.39 (3.10–3.70)
Stroke	Hypoglycemia	15	102.0 (57.1–168.3)	1.05 (0.64–1.75)	1.09 (0.66–1.81)	1.07 (0.64–1.77)
	NGT	5921	139.2 (135.7–142.8)	1	1	1
	Dysglycemia	2120	346.0 (331.4–361.1)	1.45 (1.38–1.53)	1.46 (1.38–1.53)	1.44 (1.37–1.51)
	Hyperglycemia	582	562.3 (517.6–609.9)	2.13 (1.95–2.32)	2.12 (1.94–2.31)	2.07 (1.90–2.26)
Heart failure	Hypoglycemia	4	27.0 (7.4–69.2)	0.83 (0.31–2.21)	0.54 (0.20–1.45)	0.53 (0.20–1.42)
	NGT	1982	46.3 (44.3–48.4)	1	1	1
	Dysglycemia	599	96.1 (88.6–104.1)	1.21 (1.10–1.33)	1.22 (1.11–1.34)	1.21 (1.10–1.33)
	Hyperglycemia	206	195.1 (169.3–223.6)	2.24 (1.94–2.60)	1.98 (1.71–2.30)	1.94 (1.68–2.25)

CI, confidence interval; CV, cardiovascular; HR, Hazard Ratio; n, number of patients; NGT, normal glucose tolerance; PY, patient-years; WBC, white blood cell

^a^Adjustments for: age, sex, date, hospital, and all covariates listed in Table 1, and finally adjusted for WBC

Within the first 30-days, after multiple adjustments, patients with hypoglycemia had the highest risk of all-cause mortality HR 10.87 (95% CI 8.69–13.61), followed by patients with hyperglycemia HR 7.04 (95% CI 6.50–7.63), and patients with dysglycemia HR 2.14 (95% CI 1.99–2.29), respectively, compared to patients with NGT. In contrast patients with hyperglycemia had the highest risk of cardiovascular mortality HR 16.43 (95% CI 14.36–18.80) followed by patients with hypoglycemia HR 5.29 (95% CI 2.62–10.67), and patients with dysglycemia HR 3.13 (95% CI 2.74–3.58), compared to patients with NGT (Table 2).

After multiple adjustments, the risk of myocardial infarction, stroke and heart failure was highest among patients with hyperglycemia HR 3.39 (95% CI 3.10–3.70), HR 2.07 (95% CI 1.90–2.26) and HR 1.94 (95% CI 1.68–2.25), respectively, compared to patients with NGT. Corresponding numbers for patients with dysglycemia were HR 1.70 (95% CI 1.60–1.81), HR 1.44 (95% CI 1.37–1.51), and HR 1.21 (95% CI 1.10–1.33), respectively; and for patients with hypoglycemia HR 0.77 (95% CI 0.37–1.62), HR 1.07 (95% CI 0.64–1.77), and HR 0.53 (95% CI 0.20–1.42), respectively, compared to patients with NGT (Table 2).

### Long-term outcomes—event, event rate and risk of mortality and myocardial infarction, stroke and heart failure due to blood glucose level categorization

**Fig. 1:**
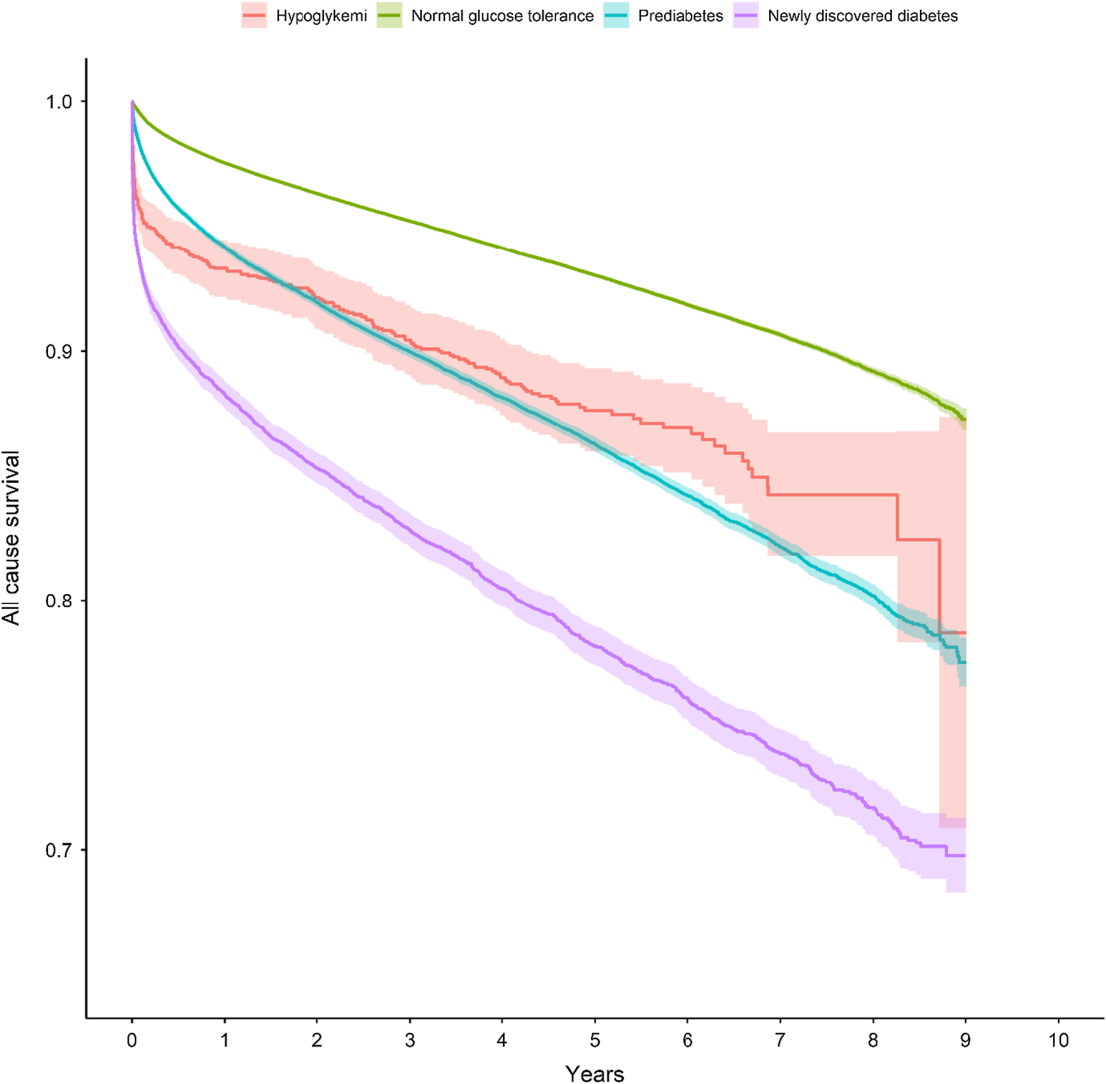
Crude estimated Kaplan–Meier curve between blood glucose and death (all-cause mortality) in 618,694 patients with previous unknown diabetes categorized into four different groups, i.e., hypoglycemia (< 3.9 mmol/L), normal glucose levels (3.9–7.7 mmol/L), dysglycemia (7.8–11.0 mmol/L) and hyperglycemia (≥ 11.1 mmol/L) according to one random glucose blood level due to visiting emergency department at seven different hospitals in Sweden between 2006 and 2016

During a mean follow-up time of 3.9 years (maximum 9 years), a total of 44,532 (7.2%) patients died: 214 (11.5%) with hypoglycemia, 31,635 (6.0%) with NGT, 9858 (12.7%) with dysglycemia, and 2825 (20.6%) patients with hyperglycemia, respectively. Events, event rate and HRs of mortality, myocardial infarction, stroke and heart failure between categorized groups are shown in Table 3. The long-term outcome of mortality is illustrated by a Kaplan Meier curve in Fig. 1. Long-term outcome of cardiovascular mortality, myocardial infarction, stroke, and hospitalization of heart failure are further illustrated in Kaplan Meier curves (Additional file 1: Fig. S1).

**Table 3:** Long term event, event rates and relative risks for all-cause mortality, cardiovascular mortality, myocardial infarction, stroke and heart failure in 618 694 patients attending the emergency department

Variables	All	Hypoglycemia < 3.9 mmol/L	NGT 3.9–7.7 mmol/L	Dysglycemia 7.8–11.1 mmol/L	Hyperglycemia > 11.1 mmol/L
n	618,694	1871	525,636	77,442	13,745
Age, Mean (SD)	47.6 (17.9)	39.6 (16.9)	46.2 (17.7)	56.1 (16.6)	57.2 (15.8)
Men, n (%)	299,151 (48.4)	778 (41.6)	247,466 (47.1)	42,510 (54.9)	8397 (61.1)
*Comorbidities*					
Hypertension, n (%)	47,340 (7.7)	105 (5.6)	36,009 (6.9)	9514 (12.3)	1712 (12.5)
Atrial fibrillation, n (%)	17,169 (2.8)	44 (2.4)	13,458 (2.6)	3127 (4.0)	540 (3.9)
Coronary heart disease, n (%)	23,272 (3.8)	52 (2.8)	17,925 (3.4)	4471 (5.8)	824 (6.0)
Prior CABG, n (%)	3015 (0.5)	6 (0.3)	2442 (0.5)	486 (0.6)	81 (0.6)
Prior PCI, n (%)	7337 (1.2)	9 (0.5)	5882 (1.1)	1245 (1.6)	201 (1.5)
Prior stroke, n (%)	11,427 (1.8)	33 (1.8)	8828 (1.7)	2170 (2.8)	396 (2.9)
Peripheral arterial disease, n (%)	1152 (0.2)	8 (0.4)	832 (0.2)	245 (0.3)	67 (0.5)
COPD, n (%)	8485 (1.4)	40 (2.1)	6378 (1.2)	1678 (2.2)	389 (2.8)
CKD, n (%)	2657 (0.4)	9 (0.5)	2060 (0.4)	487 (0.6)	101 (0.7)
^a^History of malignancy, n (%)	18,473 (3.0)	36 (1.9)	13,925 (2.6)	3813 (4.9)	699 (5.1)
*Laboratory values*					
eGFR, Mean (SD)	96.4 (21.9)	100.7 (28.5)	97.7 (21.4)	87.4 (22.5)	85.5 (26.1)
> 60 ml/min, n (%)	483,942 (94.4)	1495 (91.5)	425,360 (95.3)	49,045 (88.9)	8042 (82.8)
30–60 ml/min, n (%)	24,636 (4.8)	72 (4.4)	17,976 (4.0)	5194 (9.4)	1394 (14.3)
15–30 ml/min, n (%)	2778 (0.5)	43 (2.6)	1912 (0.4)	624 (1.1)	199 (2.0)
< 15 ml/min, n (%)	1556 (0.3)	23 (1.4)	1148 (0.3)	303 (0.5)	82 (0.8)
Random glucose, Mean (SD)	6.5 (2.6)	3.4 (0.5)	6.0 (0.8)	8.8 (0.8)	17.1 (10.7)
*Medication at index visit*					
Statin therapy, n (%)	46,885 (7.6)	65 (3.5)	35,598 (6.8)	9609 (12.4)	1613 (11.7)
Aspirin, n (%)	40,293 (6.5)	73 (3.9)	30,670 (5.8)	8150 (10.5)	1400 (10.2)
P2Y12inhibitors, n (%)	3439 (0.6)	11 (0.6)	2655 (0.5)	671 (0.9)	102 (0.7)
Betablockers, n (%)	64,023 (10.3)	132 (7.1)	48,563 (9.2)	12,916 (16.7)	2412 (17.5)
ACE/ARB, n (%)	72,244 (11.7)	98 (5.2)	55,309 (10.5)	14,347 (18.5)	2490 (18.1)
Calcium channel-blockers, n (%)	37,065 (6.0)	60 (3.2)	27,380 (5.2)	8186 (10.6)	1439 (10.5)
OAC, n (%)	14,056 (2.3)	24 (1.3)	11,046 (2.1)	2592 (3.3)	394 (2.9)
Diuretics, n (%)	32,342 (5.2)	72 (3.8)	23,945 (4.6)	6894 (8.9)	1431 (10.4)
*Socioeconomics*					
Married, n (%)	249,471 (40.3)	500 (26.7)	206,987 (39.4)	36,032 (46.5)	5952 (43.3)
Not married, n (%)	346,973 (56.1)	1333 (71.2)	301,880 (57.4)	36,788 (47.5)	6972 (50.7)
Widowed, n (%)	22,250 (3.6)	38 (2.0)	16,769 (3.2)	4622 (6.0)	821 (6.0)
*Education*					
Primary school, n (%)	131,308 (21.2)	495 (26.5)	109,027 (20.7)	17,991 (23.2)	3795 (27.6)
Collage, n (%)	254,441 (41.1)	734 (39.2)	216,009 (41.1)	31,968 (41.3)	5730 (41.7)

CI, confidence interval; CV, cardiovascular; HR, Hazard Ratio; n, number of patients; NGT, normal glucose tolerance; PY, patient-years; WBC, white blood cell

^a^Adjustments for: age, sex, date, hospital, and all covariates listed in Table 1, and finally adjusted for WBC

After multiple adjustments, the relative risk of all-cause mortality was highest among patients with hypoglycemia HR 2.58 (95% CI 2.26–2.96) followed by patients with hyperglycemia HR 1.69 (95% CI 1.63–1.76) and patients with dysglycemia HR 1.16 (95% CI 1.13–1.19), respectively, compared to the reference category of NGT. After multiple adjustments, the relative risk of cardiovascular mortality between groups was much the same as the relative risk for all-cause mortality (Table 3).

For the secondary outcomes, after multiple adjustments, the risk of myocardial infarction, stroke and heart failure were highest among patients with hyperglycemia HR 2.18 (95% CI 2.04–2.34), HR 1.54 (95% CI 1.44–1.65) and HR 1.49 (95% CI 1.36–1.63), respectively, compared to patients with NGT (Table 2). Corresponding numbers were for patients with dysglycemia HR 1.36 (95% CI 1.31–1.42), HR1.19 (95% CI 1.15–1.24) and HR 1.08 (95% CI 1.03–1.14), respectively; and for patients with hypoglycemia HR 0.89 (0.57–1.37), HR 1.14 (95% CI 0.81–1.60), and 1.02 (95% CI 0.66–1.56), respectively, compared to patients with NGT (Table 3).

After excluding the first 30-day from the analysis the results were much the same as for the main analysis (Additional file 1: Table S2).

### Sensitive analysis of early and long-term outcomes and competing risk analysis of long-term outcomes—event, event rate and risk of myocardial infarction, stroke and heart failure due to blood glucose level categorization

As the glucose levels of patients attending the ED may be influenced by acute stress, we finally adjusted our model for a marker of acute stress, i.e., WBC count, in which the relative risks of early and long-term outcomes did not change, supporting that there were no confounding effect from acute stress (Tables 2 and 3). The association between blood glucose levels and the relative risk of cardiovascular events with competing risk of death was also investigated. In a competing risk regression analysis, one could see that the sub distribution HRs for myocardial infarction, stroke and heart failure was not statistically affected after this analysis (Additional file 1: Table S3).

### Mortality and cardiovascular event rates related to sex

Event rates and risk of all-cause mortality, cardiovascular mortality, myocardial infarction, stroke and heart failure due to blood glucose level categorization in women and men, respectively, is presented in Additional file 1: Table S4. Age and sex standardized mortality rate for women was in the hypoglycemia group 57.2 (95% CI 37.5–76.9), NGT group 14.2 (95% CI 13.7–14.6), dysglycemia group 19.2 (95% CI 18.0–20.4) and hyperglycemia group 34.8 (95% CI 30.2–39.3), calculated per 1000 person-years, respectively. Corresponding numbers for men was in the hypoglycemia group 54.9 (95% CI 36.0–73.8), NGT group 20.1 (95% CI 19.5–20.6), dysglycemia group 23.5 (95% CI 22.3–24.8) and hyperglycemia group 40.5 (95% CI 36.4–44.5) calculated per 1000 person-years, respectively.

## Discussion

In this large observational study, it is shown that patients without previously known diabetes who were attending the ED at seven hospitals in Sweden between 2008 and 2016 with one random blood glucose above 7.8 mmol/L had an increased risk of death and cardiovascular events compared to patients with NGT, i.e., 3.9–7.8 mmol/L. Patients with one random blood glucose level beneath 3.9 mmol/L also had an increased risk of death compared to patients with NGT.

Previous smaller studies have found that an elevated random blood glucose in patients admitted to the ED is associated with an early mortality risk, higher readmission rates and greater length of hospital stay. The present result from this large observational study confirms earlier short-, and long-term studies [4, 7, 12–15] but also suggests that a random blood glucose level at admission predicts death and cardiovascular outcome in patients without known diabetes at a long follow-up period (maximal 9 years). It was recently demonstrated in a small, unselected cohort of patients admitted to ED, that the admission blood glucose helps to predict one-year, but not two-year mortality [16]. In the current study one random elevated blood glucose level measured in patients admitted to ED was associated to both early- and long-term increased risk of mortality and cardiovascular events. Although, most events occurred within the first year, the findings were consistent in the long run, and so were also the findings when competing risk of death was controlled for.

In the present study approximately 15% of the patients had unknown disturbances of glucose metabolism. A proportion much lower compared to earlier studies in patients admitted to the hospital due to for example myocardial infarction [17–19]. Despite a lower proportion of patients with hyperglycemia, compared to recent studies [17–19, 32, 33] the increased mortality risk and risk of cardiovascular events in patients were consistent and started already in patients categorized with mild hyperglycemia (dysglycemia). This also confirms recent studies demonstrating that even patients with mild elevated blood glucose level at hospital admission have a poorer outcome and a higher risk for cardiovascular events, compared to persons with NGT [34, 35].

Since stress hyperglycemia is common in critically ill patients, which may be observed in patients attending the ED, and is suggested as a marker of disease severity [36] we further adjusted for WBC in our final model, revealing no such confounding effect. It is known that acute illness may result in hyperglycemia due to insulin resistance caused by multiple neuroendocrine response. This response has further been suggested as an essential survival response [36]. Nevertheless, several studies have pointed toward an association between elevated random blood glucose at admission in combination with different critical ill conditions, with poor outcome and increased mortality [3, 6, 10, 37]. However, attempts to treat critically ill patients with tight glycemic control does not affect the mortality risk [38]. We cannot prove any evidence of causality between the elevated blood glucose level and risk of mortality, or cardiovascular events observed in the current study. Neither can we say that patients with one elevated blood glucose level can be categorized having diabetes or prediabetes. However, it is more likely that patients with severe hyperglycemia will be diagnosed with diabetes, i.e., mostly type 2 diabetes, in which insulin resistance occur several years before the diagnosis. Insulin resistance is multifactorial caused by genetic and environmental factors (predominantly obesity), and in a certain situation such as acute illness it may result in a reduced effect of the insulin action, which causes elevated glucose levels [39, 40]. Also a mild elevated glucose level, which may be observed in persons with insulin resistance, is a risk marker and associated with an increased risk of cardiovascular outcomes and mortality [41]. Even though stress induced hyperglycemia has been suggested an essential survival response, elevated blood glucose levels in patients in an acute situation should raise concern and always be followed-up and treated to combat cardiovascular complications [42, 43].

Only patients without a history of diabetes were included in the present study. At admission one elevated random blood glucose level was associated to not only increased mortality risk, but also to a higher incidence of myocardial infarction, stroke, and heart failure. The higher the random blood glucose level at admission, the greater was the risk of these events, both at early and at long-term follow-up. Patients with hyperglycemia at admission had more than a doubled risk of myocardial infarction, stroke and heart failure compared to patients with normal blood glucose levels. Due to the association between increased blood glucose levels at admission and the increased cardiovascular events in the long run there is reason to believe that these patients also developed diabetes. Chronic hyperglycemia is one strong, driven risk factor for the excess risk of cardiovascular events in both type 1 and type 2 diabetes over time [44, 45].

Patients admitted to the hospital for cardiovascular events often have a disturbed glucose metabolism and are usually screened by either FPG, HbA1c or OGTT to confirm diabetes or prediabetes [29]; whereas OGTT is the strongest predictor for cardiovascular events and therefore suggested the best tool in the screening for prediabetes [46, 47]. Not all persons with prediabetes will progress to type 2 diabetes; although these persons are still at higher cardiovascular risk compared to persons with NGT [41]. The risk of progression to type 2 diabetes depends on other factors such as obesity, sedentary lifestyle and sex. Lifestyle intervention and glucose lowering medication may halt this progression [48]. We do not have any information about these important factors, except for sex, in which males were at a higher absolute mortality risk compared to females. Although, the relative mortality and cardiovascular risk followed the same pattern in both sexes [49].

It is reported that critically ill patients (irrespectively of diabetes and its treatment) may have hypoglycemia [50]. This is however uncommon in adults who are not treated for diabetes, which was reflected in the present study demonstrating a very low number of patients with hypoglycemia at admission (0.3%). This group was younger, more often female and with less proportion of cardiovascular disease at baseline. After adjustment for this and other confounding factors at baseline they had the highest early- and long-term all-cause mortality risk. In patients with type 2 diabetes studies report an U-shaped association between glycemic control, i.e., HbA1c, and mortality risk especially observed in patients on insulin treatment [51]. The present study excluded patients with known diabetes, wherefore patients with hypoglycemia should not have received any insulin treatment in relation to the blood glucose level at ED admission. A more likely explanation is non-diabetes hypoglycemia which may be due to a variety of causes, e.g., hepatic, renal and cardiac failure, sepsis, trauma, burns, hormone deficiency, poisoning and malnutrition [50], making this group extra vulnerable at the ED and important to be followed-up.

### Strengths

The strength of the present study was the large study population and the long follow-up period. Other strengths of this study were its accurate determination of all-cause mortality and cardiovascular outcomes of the high quality national Swedish health data registers.

### Limitations

Our analysis was limited to admission glucose values, and we could not determine how many patients with elevated glucose on admission had persistent hyperglycemia during hospitalization or in the long run. For the same reason we could also not assess the effectiveness of early, or long-term anti-diabetes treatment, or secondary prevention, e.g., treatment against the cardiovascular outcomes of interest. As in every observational study, a possibility of residual confounding by unmeasured factors cannot be eliminated.

## Conclusion

In this large observational study including patients without known diabetes, a random blood glucose above 7.8 mmol/L at ED admission was associated with an increased early- and long-term mortality risk and cardiovascular outcome events, a risk that was further increased if blood glucose raised above 11.1 mmol/L. Patients with one random blood glucose level beneath 3.9 mmol/L had both early- and long-term increased mortality risk. This indicates that a random blood glucose in the ED can help identify patients at risk and further controlled studies to improve their outcome should be considered.

## Supplementary Information

**Additional file 1: Table S1.** ICD diagnosis used to define cardiovascular death, myocardial infarction, stroke, heart failure, percutaneous coronary intervention (PCI) and coronary artery bypass graft (CABG). **Table S2.** 30-day to 2016 event rates and relative risks for all-cause mortality, cardiovascular mortality, myocardial infarction, stroke and heart failure in 618 694 patients attending emergency department. **Table S3.** Sensitive analysis (competing risk analysis for death) of long-term cardiovascular outcomes: event rate and risk of myocardial infarction, stroke and heart failure due to blood glucose level categorization. **Table S4.** Sex stratified event, event rates and relative risks for all-cause mortality, cardiovascular mortality, myocardial infarction, stroke and heart failure in 618 694 attending the emergency department. **Table S5.** Ten most common reasons for the visit to the emergency department. **Figure S1.** Crude estimated Kaplan–Meier curves for **a)** cardiovascular mortality **b)** myocardial infarction **c)** stroke and **d)** heart failure, in 618 694 patients with previous unknown diabetes categorized into four 4 groups, i.e. hypoglycemia (< 3.9 mmol/L), normal glucose levels (3.9–7.7 mmol/L), dysglycemia (7.8–11.0 mmol/L) and hyperglycemia (≥ 11.1 mmol/L) according to one random glucose blood level due to visiting emergency department at seven different hospitals in Sweden between 2006–2016.

## Data Availability

The datasets used and/or analysed during the current study are available from the corresponding author on reasonable request. The article does not contain any individual person data in any form.
