# Correction: Admission glucose as a prognostic marker for all-cause mortality and cardiovascular disease

**DOI:** 10.1186/s12933-022-01721-3

**Published:** 2022-12-29

**Authors:** Catarina Djupsjö, Jeanette Kuhl, Tomas Andersson, Magnus Lundbäck, Martin J. Holzmann, Thomas Nyström

**Affiliations:** 1 Department of Medicine, Karolinska Institutet, Stockholm, Sweden; 2 Heart and Vascular Theme, Karolinska University Hospital, Stockholm, Sweden; 3 Division of Medicine, Danderyd University Hospital, Stockholm, Sweden; 4 Institute of Environmental Medicine, Karolinska Institutet, Stockholm, Sweden; 5 Center for Occupational and Environmental Medicine, Stockholm County Council, Stockholm, Sweden; 6 Department of Clinical Sciences, Division of Cardiovascular Medicine, Danderyd University Hospital, Karolinska Institutet, Stockholm, Sweden; 7 Functional Area of Emergency Medicine, Karolinska University Hospital, Stockholm, Sweden; 8 Department of Clinical Science and Research, Karolinska Institutet, Stockholm, Sweden; 9 Division of Internal Medicine at Södersjukhuset, Stockholm, Sweden

**Correction: Cardiovascular Diabetology (2022) 21:258** 10.1186/s12933-022-01699-y

Following publication of the original article [1], the author noticed an error in the content of Table 3.

During proof-preparation stage, the typesetter has mistakenly used the content of Table 1 instead of Table 3 which was unnoticed by the author at the time of correction process. The corrected Table 3 has been published with this erratum.

The original article has been corrected.

**Table 3:** Long term event, event rates and relative risks for all-cause mortality, cardiovascular mortality, myocardial infarction, stroke and heart failure in 618 694 patients attending the emergency department

Variable	Glucose group	Event	Event rate 1000 PY (95% CI)	Age, sex, date and hospital adjusted HR (CI 95%)	Multivariable^*^ adjusted HR (CI 95%)	Multivariable^*^ + WBC adjustments HR (CI 95%)
All–cause mortality	Hypoglycemia	214	28.8 (25.0–32.9)	3.15 (2.76–3.61)	2.64 (2.31–3.02)	2.58 (2.26–2.96)
	NGT	31 635	15.3 (15.2–15.5)	1	1	1
	Dysglycemia	9858	31.6 (31.0–32.3)	1.24 (1.21–1.27)	1.20 (1.17–1.23)	1.16 (1.13–1.19)
	Hyperglycemia	2825	53.6 (51.6–55.6)	2.04 (1.96–2.12)	1.84 (1.77–1.91)	1.69 (1.63–1.76)
CV mortality	Hypoglycemia	43	5.8 (4.2–7.8)	3.33 (2.47–4.50)	2.59 (1.91–3.49)	2.57 (1.90–3.47)
	NGT	6497	3.2 (3.1–3.2)	1	1	1
	Dysglycemia	2335	7.5 (7.2–7.8)	1.26 (1.20–1.32)	1.26 (1.20–1.32)	1.24 (1.18–1.30)
	Hyperglycemia	927	17.6 (16.5–18.8)	2.83 (2.64–3.04)	2.56 (2.38–2.74)	2.47 (2.30–2.65)
Myocardial Infarction	Hypoglycemia	20	2.7 (1.7–4.2)	1.03 (0.66–1.59)	0.93 (0.60–1.45)	0.89 (0.57–1.37)
	NGT	8535	4.2 (4.1–4.3)	1	1	1
	Dysglycemia	3030	10.0 (9.7–10.4)	1.39 (1.34–1.46)	1.39 (1.33–1.45)	1.36 (1.31–1.42)
	Hyperglycemia	940	18.7 (17.5–20.0)	2.33 (2.18–2.50)	2.28 (2.13–2.44)	2.18 (2.04–2.34)
Stroke	Hypoglycemia	34	4.6 (3.2–6.5)	1.24 (0.89–1.74)	1.19 (0.85–1.67)	1.14 (0.81–1.60)
	NGT	12 109	6.0 (5.9–6.1)	1	1	1
	Dysglycemia	3748	12.5 (12.1–12.9)	1.22 (1.17–1.27)	1.22 (1.18–1.27)	1.19 (1.15–1.24)
	Hyperglycemia	913	18.2 (17.1–19.5)	1.64 (1.53–1.76)	1.62 (1.51–1.74)	1.54 (1.44–1.65)
Heart failure	Hypoglycemia	21	2.8 (1.8–4.3)	1.46 (0.95–2.25)	1.08 (0.71–1.67)	1.02 (0.66–1.56)
	NGT	6634	3.2 (3.2–3.3)	1	1	1
	Dysglycemia	1907	6.2 (5.9–6.5)	1.11 (1.06–1.18)	1.11 (1.06–1.17)	1.08 (1.03–1.14)
	Hyperglycemia	522	10.2 (9.3–11.1)	1.75 (1.59–1.91)	1.60 (1.46–1.75)	1.49 (1.36–1.63)

CI, confidence interval; CV, cardiovascular; HR, Hazard Ratio; n, number of patients; NGT, normal glucose tolerance; PY, patient-years; WBC, White Blood Cell

l; ^*^Adjustments for: age, sex, date, hospital, and all covariates listed in Table 1, and finally adjusted for WBC
