# Supplementary material for: Admission glucose as a prognostic marker for all-cause mortality and cardiovascular disease
Source: Cardiovasc Diabetol. 2022 Nov 26;21:258. doi: 10.1186/s12933-022-01699-y (PMC9701417; doi:10.1186/s12933-022-01699-y)
Supplement: Supplementary file 1 — Additional file 1: Table S1. ICD diagnosis used to define cardiovascular death, myocardial infarction, stroke, heart failure, percutaneous coronary intervention (PCI) and coronary artery bypass graft (CABG). Table S2. 30-day to 2016 event rates and relative risks for all-cause mortality, cardiovascular mortality, myocardial infarction, stroke and heart failure in 618 694 patients attending emergency department. Table S3. Sensitive analysis (competing risk analysis for death) of long-term cardiovascular outcomes: event rate and risk of myocardial infarction, stroke and heart failure due to blood glucose level categorization. Table S4. Sex stratified event, event rates and relative risks for all-cause mortality, cardiovascular mortality, myocardial infarction, stroke and heart failure in 618 694 attending the emergency department. Table S5. Ten most common reasons for the visit to the emergency department. Figure S1. Crude estimated Kaplan–Meier curves for a) cardiovascular mortality b) myocardial infarction c) stroke and d) heart failure, in 618 694 patients with previous unknown diabetes categorized into four 4 groups, i.e. hypoglycemia (< 3.9 mmol/L), normal glucose levels (3.9–7.7 mmol/L), dysglycemia (7.8–11.0 mmol/L) and hyperglycemia (≥ 11.1 mmol/L) according to one random glucose blood level due to visiting emergency department at seven different hospitals in Sweden between 2006–2016. [file 12933_2022_1699_MOESM1_ESM.docx]

**Additional file 1**

**Admission glucose as a prognostic marker for all-cause mortality and cardiovascular disease**

Catarina Djupsjö MD^1,2^, Jeanette Kuhl MD, PhD^1,3^, Tomas Andersson^4,5^ Magnus Lundbäck MD, PhD^6,7^, Martin J. Holzmann MD, PhD^1,8^, Thomas Nyström MD, PhD^9,10^

^1^Department of Medicine, Karolinska Institutet, Stockholm, Sweden

^2^Heart and Vascular Theme, Karolinska University Hospital, Stockholm, Sweden

^3^Division of Medicine, Danderyd University Hospital, Stockholm, Sweden

^4^Institute of Environmental Medicine, Karolinska Institutet, Stockholm, Sweden

^5^Center for Occupational and Environmental Medicine, Stockholm County Council, Stockholm, Sweden

^6^Department of Clinical Sciences, Karolinska Institutet Danderyd Hospital, Stockholm, Sweden

^7^Department of Cardiology, Danderyd University Hospital, Stockholm, Sweden

^8^Functional Area of Emergency Medicine, Karolinska University Hospital, Stockholm, Sweden

^9^Department of Clinical Science and Research, Karolinska Institutet, Stockholm, Sweden

^10^Division of Internal Medicine at Södersjukhuset, Stockholm, Sweden

Table of Contents

[Table S1 – ICD diagnosis 3](#_Toc94007740)

[Table S2 – Supplementary table for 30-day - 2016 event rates and relative risks 5](#_Toc94007741)

[Table S3 - Sensitive analysis (competing risk analysis) of long-term outcomes 6](#_Toc94007742)

[Table S4 – Gender stratified event rates and relative risks 7](#_Toc94007743)

***Table S5 - Ten most common reasons for the visit to the emergency department………..…8***

[Figure S1 – Kaplan Meier curves 10](#_Toc94007744)

[Strobe- Reporting checklist for cohort study. 13](#_Toc94007747)

**Table S1.** ICD diagnosis used to define cardiovascular death, myocardial infarction, stroke, heart failure, percutaneous coronary intervention (PCI) and coronary artery bypass graft (CABG).

| **ICD diagnosis to define cardiovascular death, myocardial infarction, stroke and heart failure** | **ICD diagnosis to define PCI and CABG** |
| --- | --- |
| I05-13 | FNG02 |
| I20-21 | FNG05 |
| I24-27 | FNA00 |
| I30-31 | FNC20 |
| I33-35 | FNC10 |
| I37-38 | FNC30 |
| I40 | FNC40 |
| I42 | FNA10 |
| I44-51 | FNE96 |
| I60-I65 | FNA20 |
| I67 | FNC50 |
| I69-74 | FNE00 |
| I77 | FNE20 |
| I80-83 | FNA96 |
| I85-86 | FNC96 |
| I99 | FNC60 |
| I31 | FNC10 |
| I33 | FNB00 |
| I34 | FND96 |
| I35 | FNE10 |
| I37 | FNB20 |
| I38 | FNB96 |
| I40 | FND20 |

# Table S2. 30-day to 2016 event rates and relative risks for all-cause mortality, cardiovascular mortality, myocardial infarction, stroke and heart failure in 618 694 patients attending emergency department.

| **Variable** | **Glucose group** | **Event** | **Event rate**  **1000 PY**  **(95% CI)** | **Age, sex, date and hospital adjusted HR**  **(95% CI)** | **Multivariable adjusted HR**  **(95% CI)** |
| --- | --- | --- | --- | --- | --- |
| **All-cause mortality** | **Hypoglycemia** | 134 | 18.4 (15.4-21.8) | 2.16 (1.82-2.56) | 1.84 (1.55-2.18) |
|  | **NGT** | 29 183 | 14.5 (14.3-14.6) | 1 | 1 |
|  | **Dysglycemia** | 8 498 | 27.8 (27.2-28.4) | 1.15 (1.12-1.18) | 1.12 (1.09-1.15) |
|  | **Hyperglycemia** | 1 937 | 37.5 (35.9-39.2) | 1.51 (1.44-1.58) | 1.38 (1.32-1.45) |
| **CV mortality** | **Hypoglycemia** | 35 | 4.8 (3.3-6.7) | 3.00 (2.15-4.18) | 2.44 (1.75-3.40) |
|  | **NGT** | 5 986 | 3.0 (2.9-3.0) | 1 | 1 |
|  | **Dysglycemia** | 1 913 | 6.3 (6.0-6.5) | 1.10 (1.04-1.16) | 1.11 (1.05-1.17) |
|  | **Hyperglycemia** | 482 | 9.3 (8.5-10.2) | 1.57 (1.43-1.73) | 1.45 (1.31-1.59) |
| **Myocardial Infarction** | **Hypoglycemia** | 13 | 1.8 (1.0-3.1) | 1.29 (0.75-2.22) | 1.09 (0.63-1.89) |
|  | **NGT** | 4 711 | 2.4 (2.3-2.4) | 1 | 1 |
|  | **Dysglycemia** | 1 424 | 4.8 (4.6-5.1) | 1.14 (1.07-1.21) | 1.14 (1.07-1.21) |
|  | **Hyperglycemia** | 316 | 6.4 (5.7-7.2) | 1.42 (1.27-1.60) | 1.39 (1.24-1.56) |
| **Stroke** | **Hypoglycemia** | 19 | 2.6 (1.6-4.1) | 1.48 (0.95-2.33) | 1.36 (0.86-2.13) |
|  | **NGT** | 6 188 | 3.1 (3.0-3.2) | 1 | 1 |
|  | **Dysglycemia** | 1 628 | 5.5 (5.3-5.8) | 1.01 (0.95-1.07) | 1.01 (0.95-1.07) |
|  | **Hyperglycemia** | 331 | 6.7 (6.0-7.5) | 1.18 (1.06-1.32) | 1.16 (1.04-1.30) |
| **Heart failure** | **Hypoglycemia** | 17 | 2.3 (1.4-3.7) | 1.82 (1.13-2.92) | 1.48 (0.92-2.39) |
|  | **NGT** | 4 652 | 2.3 (2.3-2.4) | 1 | 1 |
|  | **Dysglycemia** | 1 308 | 4.3 (4.1-4.6) | 1.08 (1.01-1.15) | 1.07 (1.01-1.14) |
|  | **Hyperglycemia** | 316 | 6.3 (5.6-7.0) | 1.53 (1.36-1.72) | 1.43 (1.27-1.61) |

CI, confidence interval; CV, cardiovascular; HR, Hazard Ratio; NGT, normal glucose tolerance; PY, patient-years

Table S3. Sensitive analysis (competing risk analysis for death) of long-term cardiovascular outcomes: event rate and risk of myocardial infarction, stroke and heart failure due to blood glucose level categorization

| **Variable** | **Glucose group** | **Event rate**  **1000 PY**  **(95% CI)** | **Multivariable adjusted HR and competing risk**  **(95% CI)** |
| --- | --- | --- | --- |
| **Myocardial infarction** | **Hypoglycemia** | 2.7 (1.7-4.2) | 0.86 (0.56-1.33) |
|  | **NGT** | 4.2 (4.1-4.3) | 1 |
|  | **Dysglycemia** | 10.0 (9.7-10.4) | 1.38 (1.32-1.44) |
|  | **Hyperglycemia** | 18.7 (17.5-20.0) | 2.17 (2.02-2.33) |
| **Stroke** | **Hypglycemia** | 4.6 (3.2-6.5) | 1.10 (0.79-1.54) |
|  | **NGT** | 6.0 (5.9-6.1) | 1 |
|  | **Dysglycemia** | 12.5 (12.1-12.9) | 1.21 (1.16-1.26) |
|  | **Hyperglycemia** | 18.2 (17.1-19.5) | 1.53 (1.43-1.64) |
| **Heart failure** | **Hypoglycemia** | 2.8 (1.8-4.3) | 0.82 (0.49-1.36) |
|  | **NGT** | 3.2 (3.2-3.3) | 1 |
|  | **Dysglycemia** | 6.2 (5.9-6.5) | 1.08 (1.03-1.15) |
|  | **Hyperglycemia** | 10.2 (9.3-11.1) | 1.43 (1.30-1.58) |

CI, confidence interval; HR, Hazard Ratio; NGT, normal glucose tolerance; PY, patient-years

# Table S4. Sex stratified event, event rates and relative risks for all-cause mortality, cardiovascular mortality, myocardial infarction, stroke and heart failure in 618 694 attending the emergency department.

| **Variable** | **Glucose group** | **Event** | **Event rate**  **1000 PY**  **(95% CI)** | **Age, sex, date and hospital adjusted HR**  **(95% CI)** | **Multivariable adjusted HR**  **(95% CI)** |
| --- | --- | --- | --- | --- | --- |
| **All-cause mortality** | **Hypoglycemia** | 106 | 35.1 (28.8-42.5) | 2.68 (2.21-3.24) | 2.14 (1.77-2.59) |
| **Men** | **NGT** | 17 239 | 18.0 (17.7-18.2) | 1 | 1 |
|  | **Dysglycemia** | 5 471 | 32.0 (31.2-32.9) | 1.16 (1.12-1.20) | 1.14 (1.11-1.18) |
|  | **Hyperglycemia** | 1 650 | 51.5 (49.0-54.0) | 1.91 (1.81-2.01) | 1.77 (1.68-1.87) |
| **Women** | **Hypoglycemia** | 108 | 24.4 (20.0-29.5) | 3.82 (3.16-4.61) | 3.29 (2.72-3.98) |
|  | **NGT** | 14 396 | 13.1 (12.8-13.3) | 1 | 1 |
|  | **Dysglycemia** | 4 387 | 31.1 (30.2-32.1) | 1.35 (1.30-1.40) | 1.27 (1.22-1.31) |
|  | **Hyperglycemia** | 1 175 | 56.8 (53.6-60.2) | 2.25 (2.12-2.39) | 1.92 (1.81-2.04) |
| **CV mortality** | **Hypoglycemia** | 27 | 9.0 (5.9-13.0) | 3.11 (2.13-4.54) | 2.30 (1.57-3.36) |
| **Men** | **NGT** | 3 946 | 4.1 (4.0-4.2) | 1 | 1 |
|  | **Dysglycemia** | 1 384 | 8.1 (7.7-8.5) | 1.16 (1.09-1.23) | 1.17 (1.10-1.25) |
|  | **Hyperglycemia** | 575 | 17.9 (16.5-19.5) | 2.61 (2.39-2.85) | 2.37 (2.17-2.59) |
| **Women** | **Hypoglycemia** | 16 | 3.6 (2.1-5.9) | 3.84 (2.35-6.28) | 3.15 (1.92-5.17) |
|  | **NGT** | 2 551 | 2.3 (2.2-2.4) | 1 | 1 |
|  | **Dysglycemia** | 951 | 6.7 (6.3-7.2) | 1.43 (1.32-1.54) | 1.39 (1.28-1.50) |
|  | **Hyperglycemia** | 352 | 17.0 (15.3-18.9) | 3.20 (2.85-3.59) | 2.85 (2.54-3.20) |
| **Myocardial Infarction** | **Hypoglycemia** | 14 | 4.7 (2.6-7.9) | 0.98 (0.58-1.66) | 0.91 (0.54-1.54) |
| **Men** | **NGT** | 6 001 | 6.4 (6.2-6.5) | 1 | 1 |
|  | **Dysglycemia** | 2 120 | 12.9 (12.4-13.5) | 1.35 (1.28-1.42) | 1.35 (1.28-1.42) |
|  | **Hyperglycemia** | 665 | 22.0 (20.3-23.7) | 2.16 (2.00-2.35) | 2.14 (1.97-2.33) |
| **Women** | **Hypoglycemia** | 6 | 1.4 (0.5-3.0) | 1.22 (0.55-2.71) | 1.13 (0.51-2.53) |
|  | **NGT** | 2 534 | 2.3 (2.2-2.4) | 1 | 1 |
|  | **Dysglycemia** | 910 | 6.6 (6.2-7.0) | 1.49 (1.38-1.61) | 1.46 (1.35-1.58) |
|  | **Hyperglycemia** | 275 | 13.8 (12.2-15.5) | 2.73 (2.40-3.10) | 2.58 (2.27-2.93) |
| **Stroke** | **Hypoglycemia** | 19 | 6.4 (3.9-10.0) | 1.17 (0.75-1.84) | 1.11 (0.71-1.74) |
| **Men** | **NGT** | 6 821 | 7.3 (7.1-7.4) | 1 | 1 |
|  | **Dysglycemia** | 2 149 | 13.1 (12.5-13.6) | 1.16 (1.10-1.22) | 1.17 (1.11-1.23) |
|  | **Hyperglycemia** | 538 | 17.7 (16.2-19.2) | 1.51 (1.38-1.65) | 1.50 (1.37-1.64) |
| **Women** | **Hypoglycemia** | 15 | 3.4 (1.9-5.6) | 1.35 (0.82-2.25) | 1.34 (0.81-2.23) |
|  | **NGT** | 5 288 | 4.9 (4.7-5.0) | 1 | 1 |
|  | **Dysglycemia** | 1 599 | 11.7 (11.2-12.3) | 1.30 (1.23-1.38) | 1.29 (1.22-1.37) |
|  | **Hyperglycemia** | 375 | 19.1 (17.2-21.1) | 1.85 (1.66-2.06) | 1.81 (1.63-2.02) |
| **Heart failure** | **Hypoglycemia** | 17 | 5.7 (3.3-9.1) | 1.78 (1.10-2.86) | 1.19 (0.74-1.92) |
| **Men** | **NGT** | 4 086 | 4.3 (4.2-4.4) | 1 | 1 |
|  | **Dysglycemia** | 1 153 | 6.9 (6.5-7.3) | 1.03 (0.96-1.10) | 1.05 (0.99-1.13) |
|  | **Hyperglycemia** | 281 | 9.0 (8.0-10.1) | 1.36 (1.21-1.54) | 1.26 (1.11-1.42) |
| **Women** | **Hypoglycemia** | 4 | 0.9 (0.2-2.3) | 0.86 (0.32-2.29) | 0.86 (0.32-2.30) |
|  | **NGT** | 2 548 | 2.3 (2.2-2.4) | 1 | 1 |
|  | **Dysglycemia** | 754 | 5.4 (5.0-5.8) | 1.26 (1.16-1.37) | 1.19 (1.10-1.30) |
|  | **Hyperglycemia** | 241 | 12.0 (10.5-13.6) | 2.52 (2.20-2.89) | 2.26 (1.98-2.59) |

CI, confidence interval; CV, cardiovascular; HR, Hazard Ratio; NGT, normal glucose tolerance; PY, patient-years

Table S5. Ten most common reasons for the visit to the emergency department.

| **Search reason** | **Hypoglycemia**  **<3.9 mmol/L** | | **NGT**  **3.9-7.7 mmol/L** | **Dysglycemia**  **7.8-11.0 mmol/L** | **Hyperglycemia**  **>11.1 mmol/L** |
| --- | --- | --- | --- | --- | --- |
| **Abdominal pain, %** | | 13,4 | 17,6 | 19,9 | 13,1 |
| **Chest pain, %** | | 11,0 | 14,7 | 12,1 | 9,9 |
| **Dyspnea, %** | | 5,8 | 5,0 | 6,2 | 8,8 |
| **Headache, %** | | 9,1 | 4,9 | 2,7 | 2,2 |
| **Vertigo, %** | | 3,1 | 4,1 | 2,3 | 1,2 |
| **Syncope, %** | | 3,1 | 1,8 | 2,2 | 1,4 |
| **Stroke symptom, %** | | 1,2 | 2,3 | 3,0 | 2,8 |
| **Seizure, %** | | 2,1 | 1,1 | 1,8 | 0,9 |
| **Fever/Infection, %** | | 1,6 | 3,0 | 3,1 | 1,9 |
| **Intoxication, %** | | 5,3 | 2,0 | 2,0 | 0,7 |

Figure S1. Crude estimated Kaplan-Meier curves for **a)** cardiovascular mortality **b)** myocardial infarction **c)** stroke and **d)** heart failure, in 618 694 patients with previous unknown diabetes categorized into four 4 groups, i.e. hypoglycemia (<3.9 mmol/L), normal glucose levels (3.9-7.7 mmol/L), dysglycemia (7.8-11.0 mmol/L) and hyperglycemia (≥11.1 mmol/L) according to one random glucose blood level due to visiting emergency department at seven different hospitals in Sweden between 2006-2016.

**a)**

**
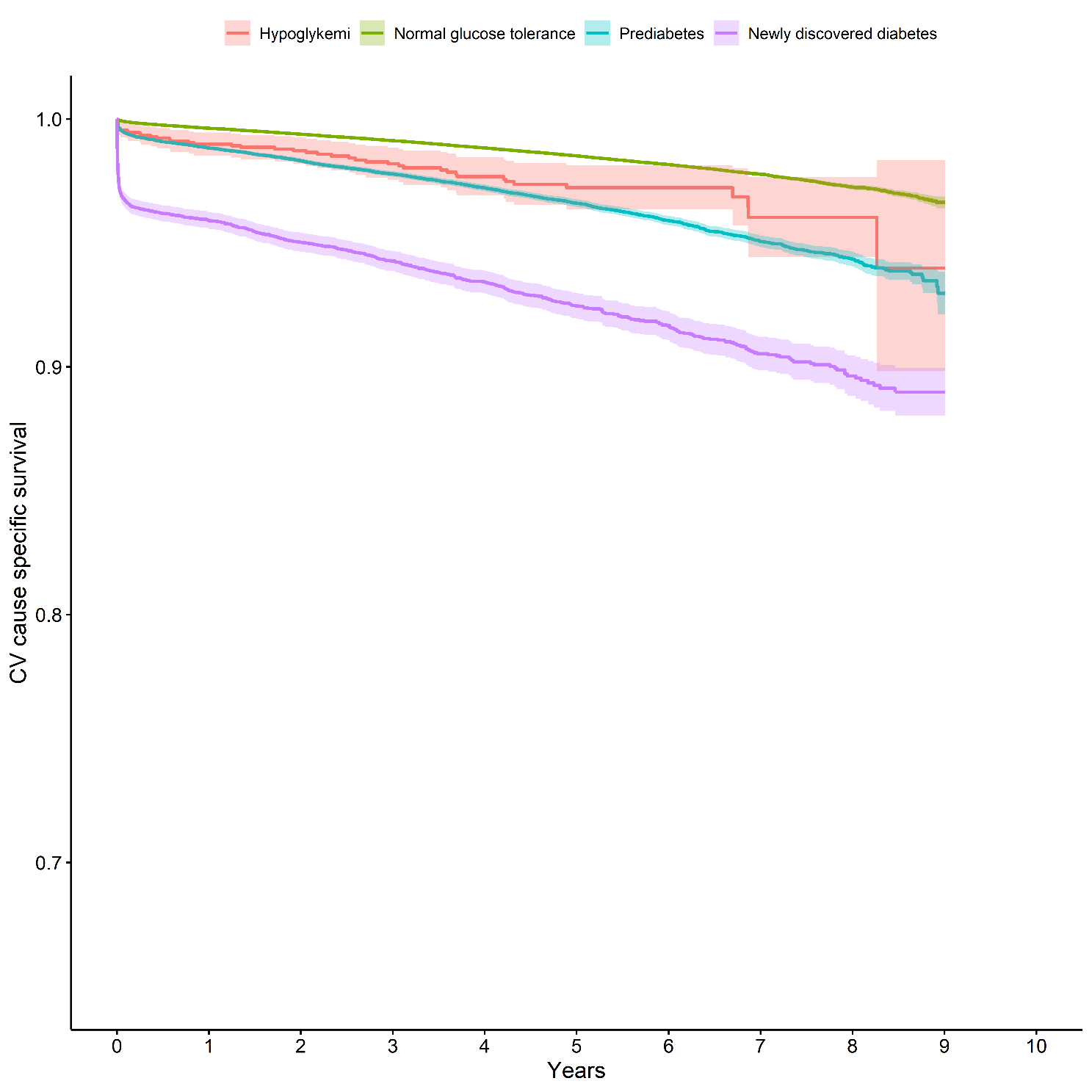
**

**b)
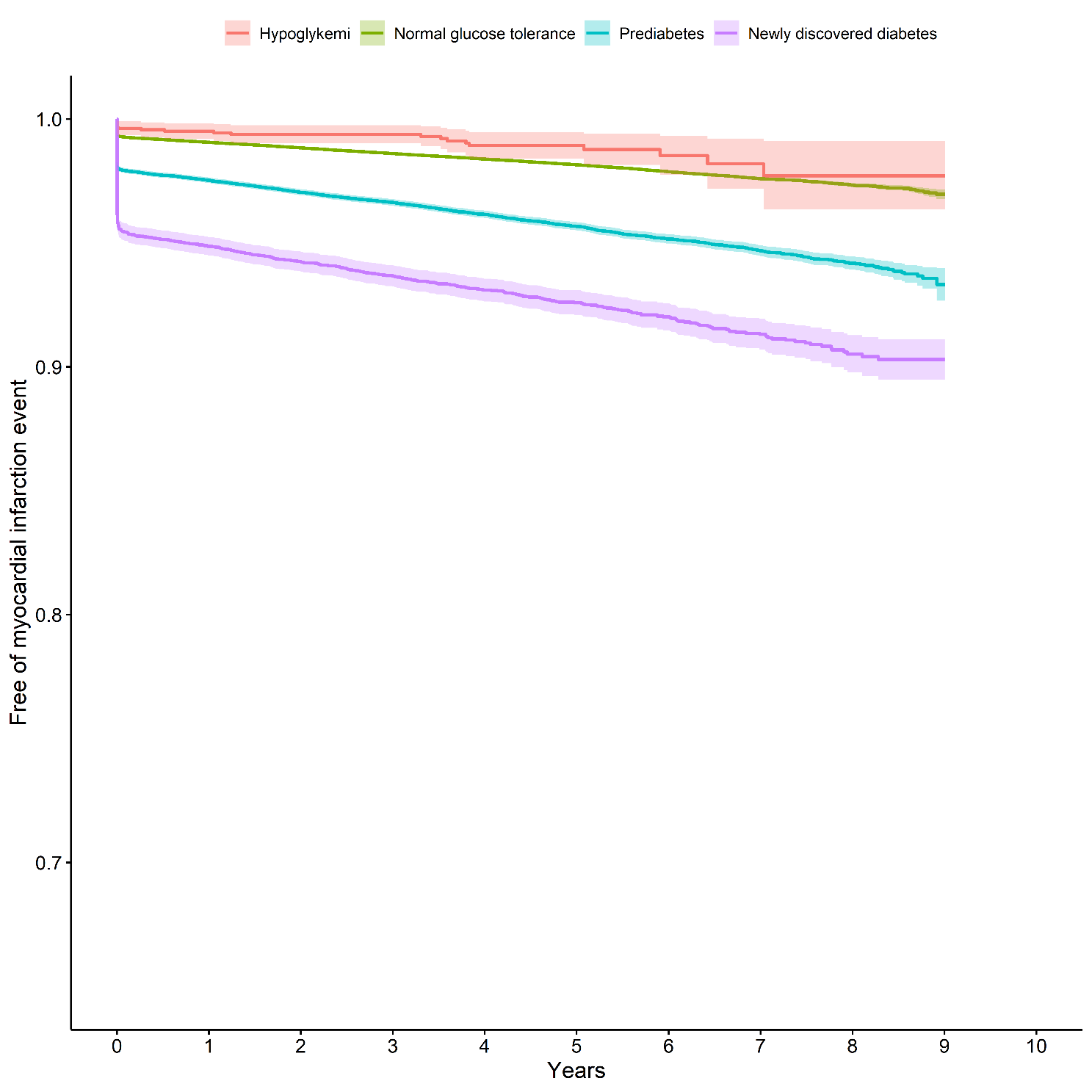
**

**c)
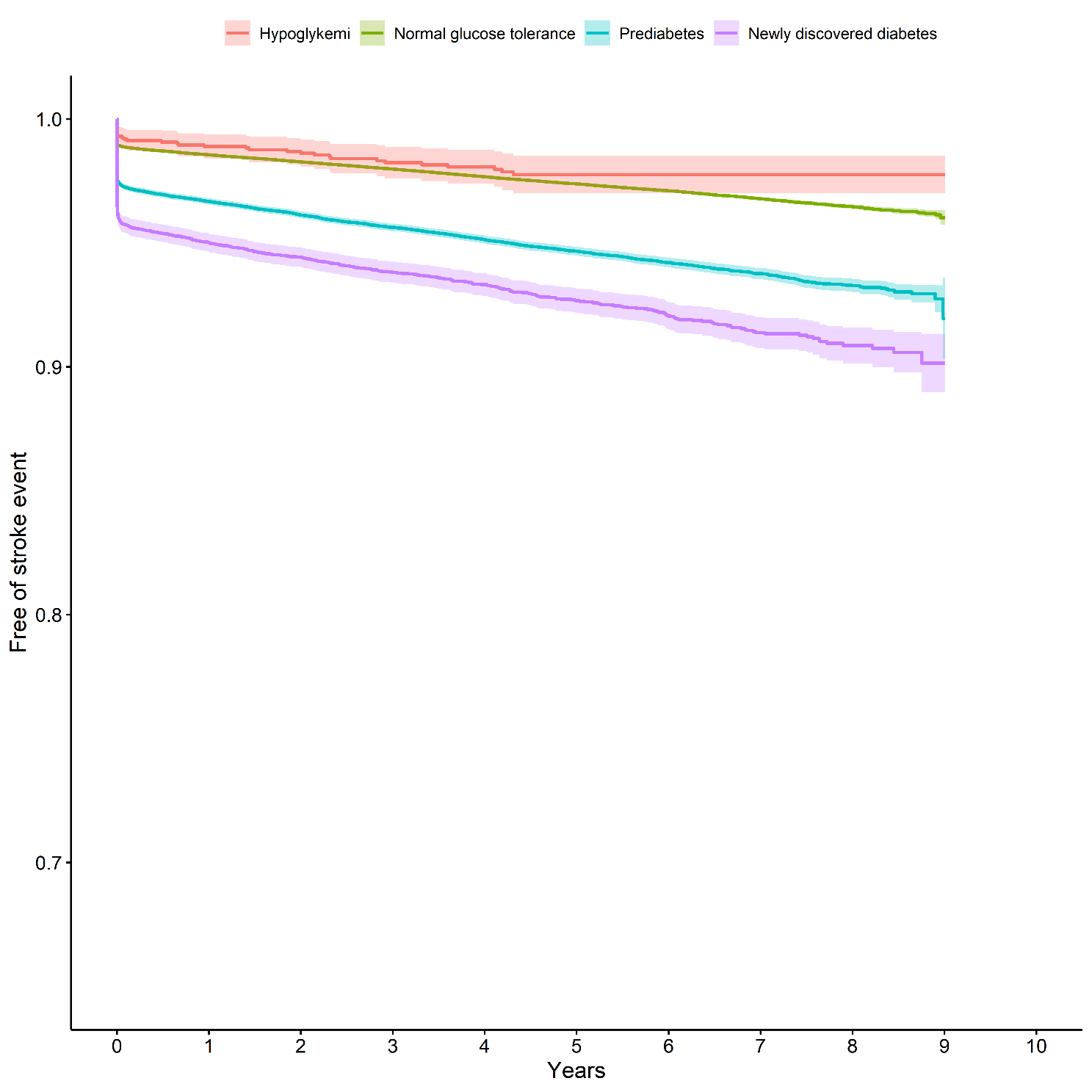
**

**d)
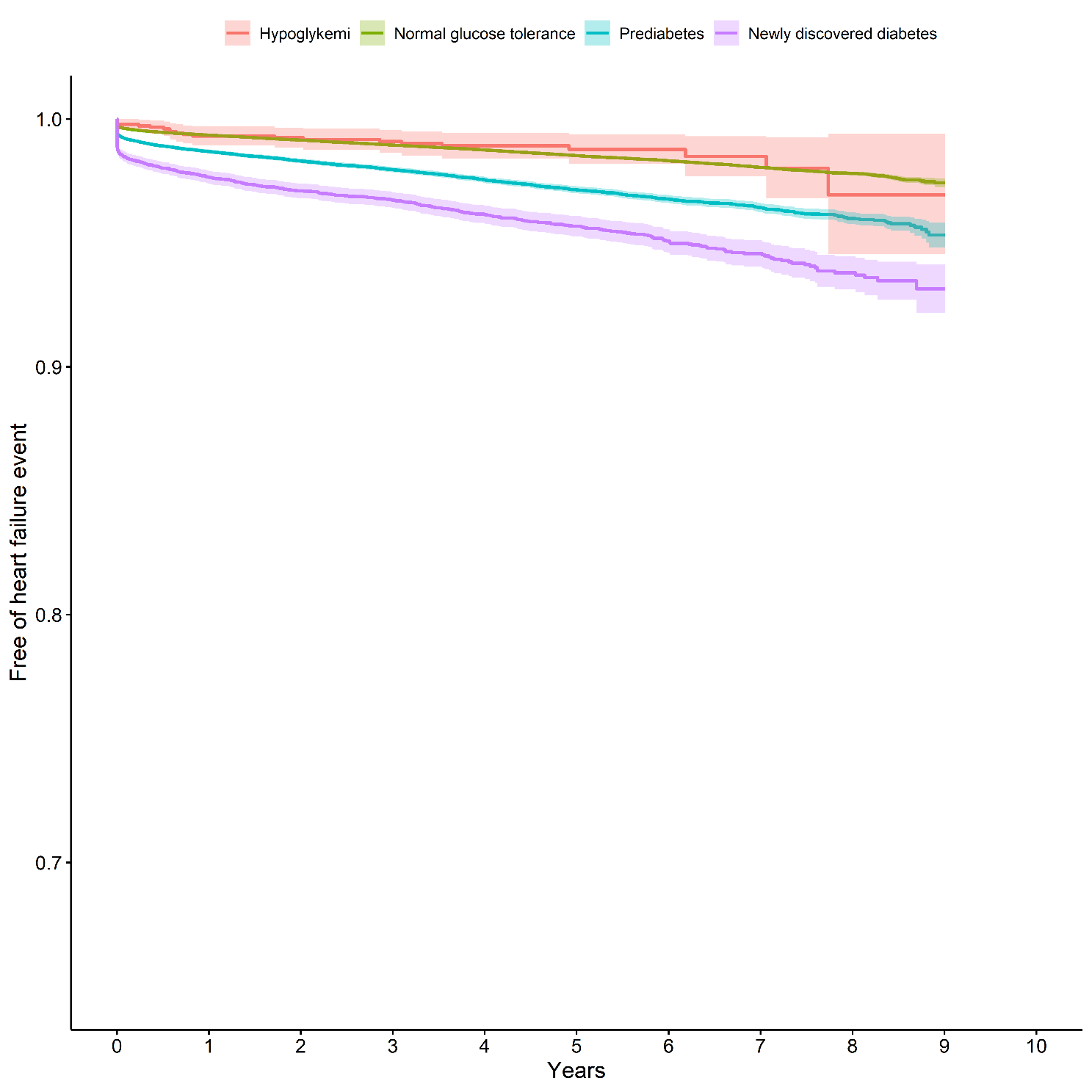
**

**Strobe checklist.** Reporting checklist for cohort study

|  |  | Reporting Item | Page Number |
| --- | --- | --- | --- |
| **Title and abstract** |  |  |  |
| Title | [#1a](https://www.goodreports.org/reporting-checklists/strobe-cohort/info/#1a) | Indicate the study’s design with a commonly used term in the title or the abstract | 1 |
| Abstract | [#1b](https://www.goodreports.org/reporting-checklists/strobe-cohort/info/#1b) | Provide in the abstract an informative and balanced summary of what was done and what was found | 2 |
| **Introduction** |  |  |  |
| Background / rationale | [#2](https://www.goodreports.org/reporting-checklists/strobe-cohort/info/#2) | Explain the scientific background and rationale for the investigation being reported | 3 |
| Objectives | [#3](https://www.goodreports.org/reporting-checklists/strobe-cohort/info/#3) | State specific objectives, including any prespecified hypotheses | 4 |
| **Methods** |  |  |  |
| Study design | [#4](https://www.goodreports.org/reporting-checklists/strobe-cohort/info/#4) | Present key elements of study design early in the paper | 5-6 |
| Setting | [#5](https://www.goodreports.org/reporting-checklists/strobe-cohort/info/#5) | Describe the setting, locations, and relevant dates, including periods of recruitment, exposure, follow-up, and data collection | 5-6 |
| Eligibility criteria | [#6a](https://www.goodreports.org/reporting-checklists/strobe-cohort/info/#6a) | Give the eligibility criteria, and the sources and methods of selection of participants. Describe methods of follow-up. | 5-7 |
| Eligibility criteria | [#6b](https://www.goodreports.org/reporting-checklists/strobe-cohort/info/#6b) | For matched studies, give matching criteria and number of exposed and unexposed | 5-7 |
| Variables | [#7](https://www.goodreports.org/reporting-checklists/strobe-cohort/info/#7) | Clearly define all outcomes, exposures, predictors, potential confounders, and effect modifiers. Give diagnostic criteria, if applicable | 5-7 |
| Data sources / measurement | [#8](https://www.goodreports.org/reporting-checklists/strobe-cohort/info/#8) | For each variable of interest give sources of data and details of methods of assessment (measurement). Describe comparability of assessment methods if there is more than one group. Give information separately for exposed and unexposed groups if applicable. | 5-7 |
| Bias | [#9](https://www.goodreports.org/reporting-checklists/strobe-cohort/info/#9) | Describe any efforts to address potential sources of bias | 5-7 |
| Study size | [#10](https://www.goodreports.org/reporting-checklists/strobe-cohort/info/#10) | Explain how the study size was arrived at | 5 |
| Quantitative variables | [#11](https://www.goodreports.org/reporting-checklists/strobe-cohort/info/#11) | Explain how quantitative variables were handled in the analyses. If applicable, describe which groupings were chosen, and why | 5-7 |
| Statistical methods | [#12a](https://www.goodreports.org/reporting-checklists/strobe-cohort/info/#12a) | Describe all statistical methods, including those used to control for confounding |  |
| 7 |  |  |  |
| Statistical methods | [#12b](https://www.goodreports.org/reporting-checklists/strobe-cohort/info/#12b) | Describe any methods used to examine subgroups and interactions | 7 |
| Statistical methods | [#12c](https://www.goodreports.org/reporting-checklists/strobe-cohort/info/#12c) | Explain how missing data were addressed | 7 |
| Statistical methods | [#12d](https://www.goodreports.org/reporting-checklists/strobe-cohort/info/#12d) | If applicable, explain how loss to follow-up was addressed | 7 |
| Statistical methods | [#12e](https://www.goodreports.org/reporting-checklists/strobe-cohort/info/#12e) | Describe any sensitivity analyses |  |
| 7 |  |  |  |
| **Results** |  |  |  |
| Participants | [#13a](https://www.goodreports.org/reporting-checklists/strobe-cohort/info/#13a) | Report numbers of individuals at each stage of study—e.g. numbers potentially eligible, examined for eligibility, confirmed eligible, included in the study, completing follow-up, and analyzed. Give information separately for exposed and unexposed groups if applicable. | 8-10 |
| Participants | [#13b](https://www.goodreports.org/reporting-checklists/strobe-cohort/info/#13b) | Give reasons for non-participation at each stage | 5 |
| Participants | [#13c](https://www.goodreports.org/reporting-checklists/strobe-cohort/info/#13c) | Consider use of a flow diagram |  |
| 5 |  |  |  |
| Descriptive data | [#14a](https://www.goodreports.org/reporting-checklists/strobe-cohort/info/#14a) | Give characteristics of study participants (e.g. demographic, clinical, social) and information on exposures and potential confounders. Give information separately for exposed and unexposed groups if applicable. | 8-10 |
| Descriptive data | [#14b](https://www.goodreports.org/reporting-checklists/strobe-cohort/info/#14b) | Indicate number of participants with missing data for each variable of interest |  |
| table 1 |  |  |  |
| Descriptive data | [#14c](https://www.goodreports.org/reporting-checklists/strobe-cohort/info/#14c) | Summarize follow-up time (e.g., average and total amount) |  |
| 8 |  |  |  |
| Outcome data | [#15](https://www.goodreports.org/reporting-checklists/strobe-cohort/info/#15) | Report numbers of outcome events or summary measures over time. Give information separately for exposed and unexposed groups if applicable. |  |
| 8-10 |  |  |  |
| Main results | [#16a](https://www.goodreports.org/reporting-checklists/strobe-cohort/info/#16a) | Give unadjusted estimates and, if applicable, confounder-adjusted estimates and their precision (e.g., 95% confidence interval). Make clear which confounders were adjusted for and why they were included | 8-10 |
| Main results | [#16b](https://www.goodreports.org/reporting-checklists/strobe-cohort/info/#16b) | Report category boundaries when continuous variables were categorized | 8-10 |
| Main results | [#16c](https://www.goodreports.org/reporting-checklists/strobe-cohort/info/#16c) | If relevant, consider translating estimates of relative risk into absolute risk for a meaningful time period |  |
| 8-10 |  |  |  |
| Other analyses | [#17](https://www.goodreports.org/reporting-checklists/strobe-cohort/info/#17) | Report other analyses done—e.g. analyses of subgroups and interactions, and sensitivity analyses | 8-10 |
| **Discussion** |  |  |  |
| Key results | [#18](https://www.goodreports.org/reporting-checklists/strobe-cohort/info/#18) | Summarize key results with reference to study objectives | 12-14 |
| Limitations | [#19](https://www.goodreports.org/reporting-checklists/strobe-cohort/info/#19) | Discuss limitations of the study, taking into account sources of potential bias or imprecision. Discuss both direction and magnitude of any potential bias. | 12-14 |
| Interpretation | [#20](https://www.goodreports.org/reporting-checklists/strobe-cohort/info/#20) | Give a cautious overall interpretation considering objectives, limitations, multiplicity of analyses, results from similar studies, and other relevant evidence. | 12-14 |
| Generalizability | [#21](https://www.goodreports.org/reporting-checklists/strobe-cohort/info/#21) | Discuss the generalizability (external validity) of the study results | 12-14 |
| **Other Information** |  |  |  |
| Funding | [#22](https://www.goodreports.org/reporting-checklists/strobe-cohort/info/#22) | Give the source of funding and the role of the funders for the present study and, if applicable, for the original study on which the present article is based | 15 |

The STROBE checklist is distributed under the terms of the Creative Commons Attribution License CC-BY. This checklist was completed on 22. November 2021 using <https://www.goodreports.org/>, a tool made by the [EQUATOR Network](https://www.equator-network.org) in collaboration with [Penelope.ai](https://www.penelope.ai)
